# Assessment of the Oval Window–Facial Canal Corridor and Mastoid Pneumatization: A Preliminary HRCT-Based Radiological–Anatomical Framework

**DOI:** 10.3390/diagnostics16142200

**Published:** 2026-07-14

**Authors:** Burak Bilecenoğlu, Tuğçe Akın, Berin Tuğtağ Demir, Hilal Melis Altıntaş, Ali Köksal, Süha Beton

**Affiliations:** 1 Department of Anatomy, Faculty of Medicine, Ankara Medipol University, Ankara 06570, Turkey; tugce.akin@ankaramedipol.edu.tr (T.A.); berin.tugtag@ankaramedipol.edu.tr (B.T.D.); hilal.altintas@ankaramedipol.edu.tr (H.M.A.); 2 Department of Medical Services and Techniques, Vocational School of Health Services, Atılım University, Ankara 06530, Turkey; akoksal@bayindirhastanesi.com.tr; 3 Department of Radiology, Private Acıbadem Bayındır Hospital, Ankara 06250, Turkey; 4 Department of Ear, Nose, Throat Surgery, Faculty of Medicine, Ankara University, Ankara 06230, Turkey; sbeton@ankara.edu.tr

**Keywords:** oval window, facial nerve, mastoid pneumatization, computed tomography

## Abstract

**Background/Objectives**: The anatomical relationship between the oval window (OW) and the facial nerve (FN) is of significant importance in the context of stapes and middle ear surgery. However, the influence of mastoid pneumatization on OW morphology and FN proximity remains to be elucidated. **Methods**: This retrospective study included 220 radiologically normal temporal bones from 110 adults. OW width, OW height, OW niche width, FNt–OW distance, FNm–OW distance, OW–mastoid cell distance, and OW–FNm–FNt corridor area were measured on HRCT images. Mastoid pneumatization was classified using a sigmoid sinus-based grading system. RASCI was developed by integrating OW morphology, OW niche width, and mastoid pneumatization. **Results:** The mean width of the OW, the height of the OW, and the width of the OW niche were 2.41 ± 0.92 mm, 1.38 ± 0.36 mm, and 1.03 ± 0.38 mm, respectively. The mean FNt–OW and FNm–OW distances were recorded as 0.93 ± 0.33 mm and 2.78 ± 0.94 mm, respectively. It was established that larger OW dimensions and FN-OW distances were associated with higher levels of mastoid pneumatization. The radiological–anatomical constraint was categorised as low in 21.4% of cases, moderate in 69.5%, and high in 9.1% of the temporal bones (RASCI). **Conclusions**: Mastoid pneumatization appears to be associated with OW morphology and FN–OW spatial relationships. The RASCI could provide an initial HRCT-based framework for grading the constraints of the OW-FN corridor; however, prospective surgical validation is required.

## 1. Introduction

The complex spatial relationship between the oval window (OW) and the tympanic (FNt) and the mastoid (FNm) segments of the facial nerve represents one of the most critical anatomical corridors in otologic and skull base surgery [1,2,3,4]. The OW is the primary gateway to the vestibule; as such, it is pivotal to stapes surgery performed in cases of otosclerosis [5,6,7]. Furthermore, its morphology and relationship with the facial nerve are clinically relevant in middle-ear procedures involving the oval-window region, including ossicular chain reconstruction, tympanoplasty, and selected transcanal or endoscopic approaches to the medial tympanic wall [8,9,10]. The narrow surgical field of view, coupled with the high fragility of adjacent neurovascular structures, renders even minor anatomical variations within the oval window niche (OWn) or an unexpected course of the facial nerve capable of dramatically increasing the risk of iatrogenic facial paralysis or inadequate ossicular reconstruction [3,11,12,13].

The morphometric dimensions of the oval window floor directly determine the selection, optimal placement, and long-term auditory success of stapes prostheses [5,6,7]. The limits of surgical manipulation within this narrow corridor are strictly defined by the complex topography of the facial nerve (FN) [1,3,11]. While the tympanic segment of the FN, which courses immediately superior to the oval window, is recognized as the most vulnerable portion for facial canal dehiscence, the vertical mastoid segment delineates the posterior limits of the surgical approach [14,15,16]. Iatrogenic FN injury, although uncommon, remains one of the most feared complications of stapes and middle ear surgery, particularly in revision cases or in the presence of dehiscent, prolapsed, or overhanging facial nerve anatomy [3,12,13]. Moreover, operating within a restricted OW surgical corridor, characterized by limited three-dimensional spatial perception, may increase the risk of acoustic trauma, perilymphatic fistula, and postoperative sensorineural hearing loss [11,17].

Although the isolated morphometric dimensions of the OW and FN have been extensively studied in previous cadaveric studies, the dynamic spatial interactions between these structures in the living human temporal bone have not yet been sufficiently optimised in preoperative radiological planning [1,6,7,14,17]. Specifically, the morphological implications of the temporal bone’s overall degree of pneumatization (mastoid pneumatization) on the microsurgical corridor extending to the OW represent a critical gap in the literature [18,19,20,21]. Although previous radiological studies have shown that temporal bone pneumatization is associated with variations in sigmoid sinus position and facial canal-related measurements, its direct modulatory effect on OW morphology and FN–OW proximity has not yet been quantitatively mapped using HRCT [19,20,21,22].

Based on the existing literature, hypopneumatized temporal bones may be expected to present narrower surgical corridors and altered facial canal relationships, potentially increasing the complexity of OW-related procedures [19,20,21,22]. Furthermore, the present study proposes that these anatomical constraints may be better described not only through isolated linear measurements but also through an integrated radiological–anatomical framework that may support structured preoperative assessment [16,17]. The objective of this study was to evaluate the morphometric relationships between the oval window, the oval window niche, and the tympanic/mastoid segments of the facial nerve. In addition, this study explored how these parameters varied according to different degrees of mastoid pneumatization. A further objective was to develop a novel HRCT-based Radiological–Anatomical Surgical Corridor Index (RASCI) model. The proposed model may help stratify radiological–anatomical difficulty by combining the parameters of mastoid pneumatization, oval window width, and facial nerve distance. Finally, the potential clinical applicability of this model in preoperative surgical planning was discussed in light of the existing literature.

## 2. Materials and Methods

### 2.1. Ethics and Study Population

The medical ethics committee of Bayındır Söğütözü Hospital approved this retrospective, single-center study, and the requirement for informed consent was waived owing to the retrospective design of the study (BTEDK 25/05). Between May 2023 and May 2025, temporal bone CT images were retrospectively reviewed.

### 2.2. Clinical Indications and Ear Selection

All CTs were originally performed for routine clinical indications, primarily cochlear implant planning, evaluation of chronic otitis media in the contralateral ear, and trauma assessment. Although CT examinations were obtained for different clinical indications, only ears without radiological evidence of middle-ear or inner-ear pathology were included in the analysis. This ensured that the analysis of 220 ears from 110 patients was based exclusively on radiologically normal temporal bones.

The inclusion criteria were as follows:

(1) be aged 18 years or over;
(2) not present with any symptoms of FN dysfunction or cranial lesion;
(3) ears without any evidence of middle-ear or inner-ear pathology (e.g., cholesteatoma, mastoiditis, ossicular erosion) on CT. Images of insufficient quality (because of the presence of motion artifacts or incomplete images) were excluded from the study.

### 2.3. Image Acquisition

The images were obtained with a 32-detector multi-slice CT device with a section thickness of 0.625 mm in the axial plane. Two researchers, one of whom is a radiologist and an anatomist with expertise in cross-sectional anatomy, and the other is also a radiologist and clinical anatomist, evaluated images of 220 temporal bones belonging to 110 patients. All images were exported in DICOM file format and archived in a three-dimensional workstation using dedicated software (RadiAnt DICOM Viewer, RadiAnt, Poznan, Poland, Version 2025.1) for subsequent image analysis.

### 2.4. Image Analysis

**Figure 1:**
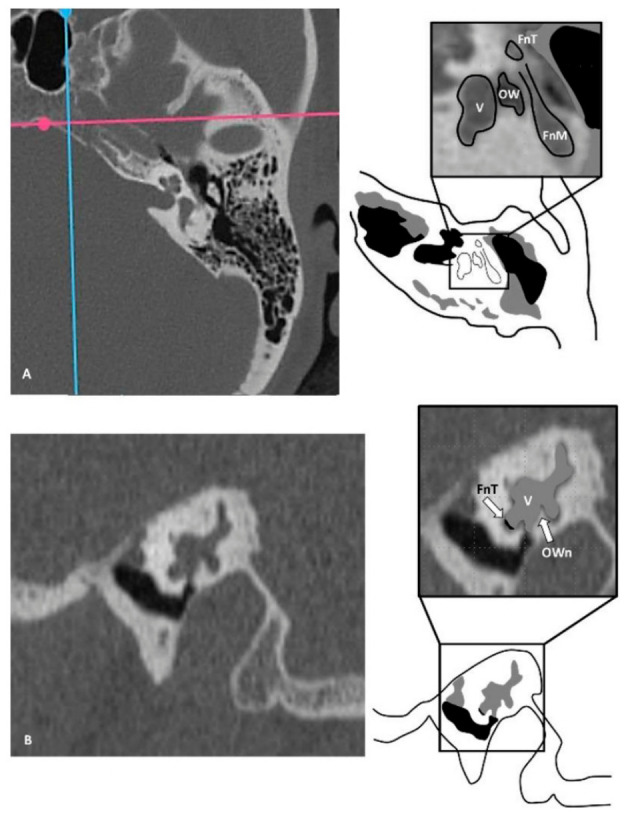
(**A**) Measurement of the OW and FN on an axial plane (Pink line: coronal plane; Blue line: sagittal plane); (**B**) Measurement of the OWn on an axial plane. (OW: Oval window, OWn: Oval window niche, FNt: Tympanic segment of the facial nerve, FNm: Mastoid segment of the facial nerve, V: Vestibule, FN: Facial nerve).

Following multiplanar reconstruction, standardized axial and oblique planes were obtained to visualize the OW, OWn, FNt, and FNm. Window width and level settings were adjusted to optimize visualization of the bony margins of the OW, OWn, and facial canal. It is important to note that all measurements were performed separately for the right and left temporal bones. In addition, it was decided that bilateral values would not be averaged, and that each temporal bone would be analysed as an individual radiological unit. The measurements were obtained on the basis of standardised axial or oblique axial multiplanar reconstructions, where the relevant bony anatomical landmarks were most clearly visualised, using bone-window settings. The width of the OW was measured as the maximum anteroposterior diameter of the bony oval window rim, and the height was measured perpendicular to this diameter on the same reconstructed plane. As the facial nerve itself cannot be directly visualised on HRCT, FN-related measurements were performed using the bony margins of the facial canal. The FNt–OW distance was defined as the shortest linear distance from the inferior bony margin of the tympanic segment of the facial canal to the superior bony rim of the oval window. The FNm–OW distance was defined as the shortest linear distance from the anteromedial bony margin of the mastoid segment of the facial canal to the nearest posterior or posterolateral bony rim of the oval window. The measurement of the OW niche width was taken as the minimum distance between the opposing bony walls of the oval window niche at its narrowest visible portion, on the reconstructed plane in which the oval window niche and footplate region were most clearly delineated (Figure 1).

#### 2.4.1. Radiological–Anatomical Surgical Corridor Index (RASCI)

For clarity, in the present study, OW width was defined as the maximum anteroposterior diameter of the oval window, whereas OW height was defined as the diameter measured perpendicular to this anteroposterior axis on the same reconstructed plane. It should be noted that previous studies may have used different terminology for comparable dimensions; therefore, the thresholds used in the RASCI were adapted according to the measurement definitions applied in the present study. The RASCI was designed as a preliminary ordinal radiological–anatomical framework rather than a validated clinical prediction score. The thresholds and point values were selected a priori based on previously reported anatomical/radiological cut-offs where available and on the expected direction of anatomical constraint within the OW–FN corridor. Because the present study did not include intraoperative difficulty grading or postoperative outcome data, data-driven weighting or ROC-based cut-off optimization could not be performed.

Parameter 1: Oval Window (OW) Height: OW height, corresponding to the perpendicular/vertical dimension of the oval window in the present study, was categorized using thresholds derived from previous radiological and anatomical studies evaluating restricted oval window access [5,17].

- <0.8 mm: 3 points, marked radiological–anatomical constraint
- 0.8–1.4 mm: 2 points, moderate radiological–anatomical constraint
- ≥1.4 mm: 1 point, low radiological–anatomical constraint

Parameter 2: Oval Window (OW) Width: OW width, defined in the present study as the maximum anteroposterior diameter of the oval window, was categorized using a 1.0-mm threshold to identify markedly reduced anteroposterior dimensions and a 2.0-mm threshold to define relatively wider configurations [17].

- <1.0 mm: 3 points, marked constraint
- 1.0–2.0 mm: 2 points, moderate constraint
- >2.0 mm: 1 point, low constraint

Parameter 3: Degree of Mastoid Pneumatization: Mastoid pneumatization was classified according to Han et al.’s sigmoid sinus-based CT grading system [22]. In the present study, higher grades of pneumatization were associated with larger OW dimensions and greater FN–OW distances. Therefore, hypopneumatized temporal bones were considered to represent a more spatially constrained radiological–anatomical configuration within the proposed index.

- Grade I: 3 points, hypopneumatized configuration
- Grade II: 2 points, intermediate pneumatization
- Grades III–IV: 1 point, well-pneumatized configuration

Parameter 4: Oval Window (OW) Niche Width: OW niche width was incorporated into the index because a narrow OW niche may impose additional spatial constraints during OW-related procedures [11,17].

- <1.4 mm: 2 Points, narrow niche configuration
- ≥1.4 mm: 1 Point, relatively wider niche configuration

#### 2.4.2. Calculating the Total Radiological–Anatomical Surgical Corridor Index

The total RASCI score was calculated by summing the four parameter scores, yielding a range from 4 to 11 points for each temporal bone. The total score was then categorized into three radiological–anatomical constraint groups:

- Total 4–5 Points (Stage 1/Low Radiological–Anatomical Constraint): The mastoid is well-ventilated (Type III–IV), and the OW width and height are within relatively favorable radiological thresholds (>1.4 mm and >2.0 mm). This category may represent a favorable radiological–anatomical configuration, characterized by wider OW dimensions and greater FN–OW distances. However, its relationship with actual intraoperative difficulty or complication rates requires clinical validation.
- Total 6–8 Points (Stage 2/Moderate Radiological–Anatomical Constraint): This category includes temporal bones with intermediate mastoid pneumatization and borderline OW or OW niche dimensions. These findings may indicate a moderately constrained OW–FN corridor and may support more detailed preoperative HRCT assessment.
- Total 9–11 Points (Stage 3/High Radiological–Anatomical Constraint): Hypopneumatic mastoid (Type I), very narrow oval window (<0.8 mm), and narrow oval window niche. This category may indicate a potentially narrow OW–FN surgical corridor, characterized by reduced OW dimensions and closer FN proximity. In such cases, the RASCI may prompt more detailed preoperative assessment, careful instrument selection, or consideration of alternative technical strategies, depending on the surgeon’s intraoperative judgment.

#### 2.4.3. Evaluation of Mastoid Pneumatization

**Figure 2:**
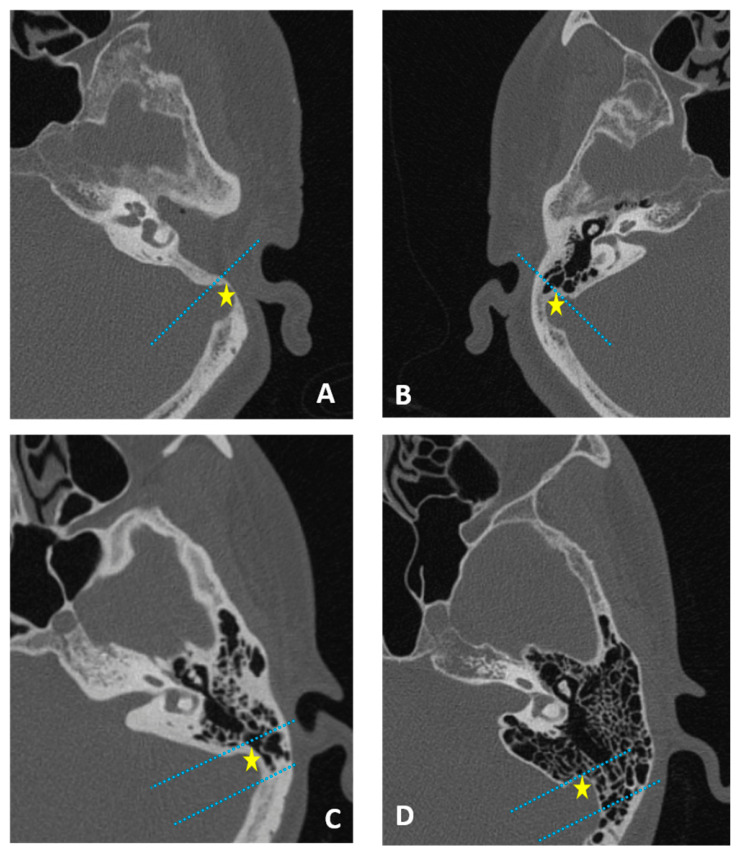
Han et al.’s sigmoid sinus-based classification of mastoid pneumatization on an axial plane (Star symbol: sigmoid sinus; Dash lines: anterior, medial and posterior borders of the sigmoid sinus). (**A**) Grade I: pneumatization anterior/anteromedial to the line through the most anterior point of the sigmoid sinus. (**B**) Grade II: pneumatization between the anterior and lateral sigmoid sinus lines. (**C**) Grade III: pneumatization between the lateral and posterior sigmoid sinus lines. (**D**) Grade IV: pneumatization extending beyond the posterior sigmoid sinus line.

Mastoid pneumatization was classified according to the extent of mastoid air-cell development in relation to the sigmoid sinus, using the method described by Han et al. [22]. On axial CT imaging, the extent of pneumatization was categorized according to a grading system. Grade I was assigned when mastoid pneumatization was confined to the anteromedial region of the arbitrary line passing through the most anterior point of the sigmoid sinus. Grade II was assigned when pneumatization was located between the two arbitrary lines passing through the most anterior point and the most lateral aspect of the sigmoid sinus. Grade III was assigned when pneumatization was located between the two arbitrary lines passing through the most lateral aspect and the most posterior point of the sigmoid sinus. Grade IV was assigned when pneumatization extended beyond the arbitrary line passing through the most posterior point of the sigmoid sinus (Figure 2).

### 2.5. Statistical Analysis

The statistical analysis was conducted using IBM SPSS Statistics, version 22.0. The Kolmogorov–Smirnov test was employed to ascertain the normality of the distribution of variables. Continuous variables were summarized as mean ± SD for descriptive purposes. Because several variables showed non-normal distribution, between-group comparisons were performed using non-parametric tests. Inter-rater repeatability was assessed using a two-way random-effects, absolute-agreement intra-class correlation coefficient (ICC), which was 0.92 (95% CI 0.88–0.95). The between-group differences regarding the continuous variables were assessed using the Kruskal–Wallis test (post hoc Dunn’s test). The correlations between the parameters of the OW, FN, and mastoid pneumatization were examined with Spearman’s Rho correlation coefficient test. The statistical significance level was set at 0.05.

## 3. Results

A total of 110 patients (220 temporal bones) were included in the study, comprising 58 males (52.7%) and 52 females (47.3%). The mean age of the participants was 45 ± 3.56 years. The mean OW width was 2.41 ± 0.92 mm, the mean OW height was 1.38 ± 0.36 mm, and the mean OW niche width was 1.03 ± 0.38 mm. The distance between the FNt and OW was 0.93 ± 0.33 mm, while the distance between the FNm and OW was 2.78 ± 0.94 mm. The area between the FNm, FNt, and OW was calculated to be 14.0 ± 1.0 mm^2^ (Table 1).

**Figure 3:**
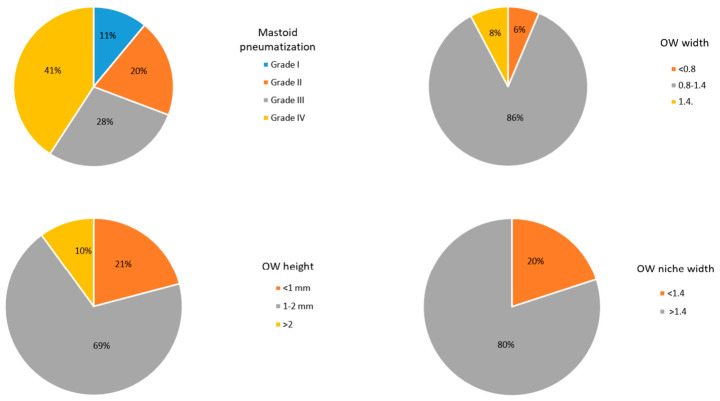
Mastoid pneumatization and oval window classification.

Mastoid pneumatization was observed to be grade I in 10.9% (n = 24) of the temporal bones, grade II in 19.5% (n = 43), grade III in 28.2% (n = 62), and grade IV in 41.4% (n = 91). The OW height was <0.8 mm in 6.4% (n = 14) of cases, while the OW width was <1.0 mm in 20.9% (n = 46) of cases (Figure 3) (Table 2).

Significant between-group differences were observed in OW width, OW area, FNt–OW distance, FNm–OW distance, and OW–FNm–FNt area across OW height categories (*p* < 0.05) (Table 3).

**Figure 4:**
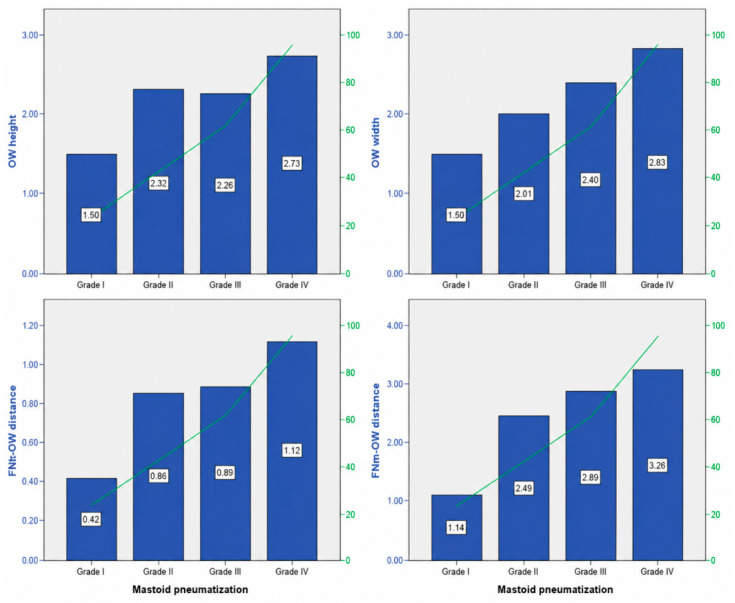
Mastoid pneumatization in relation to oval window and facial nerve. The green line represents the cumulative percentage of cases across the mastoid pneumatization grades, as indicated by the right *y*-axis.

A statistically significant correlation was observed between mastoid pneumatization and OW width and height. As the degree of pneumatization increased, FNt–OW and FNm–OW distances also increased (*p* < 0.05) (Figure 4).

Larger OW dimensions were generally associated with greater FN–OW distances. As OW width, height, and niche width increased, FNt-OW distance and FNm-OW distance also increased (Table 4).

**Figure 5:**
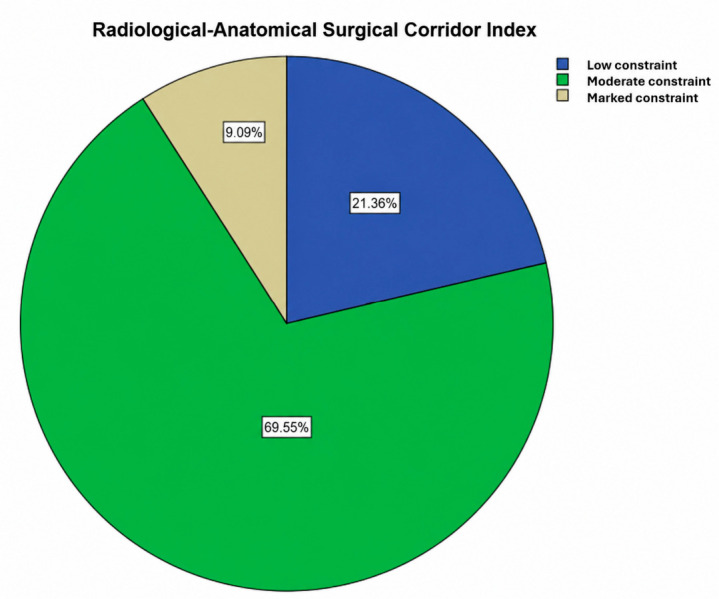
Distribution of temporal bones according to the Radiological–Anatomical Surgical Corridor Index (RASCI) categories.

According to the preliminary RASCI classification, 47 temporal bones (21.4%) were categorized as having low radiological–anatomical constraint, 153 (69.5%) as having moderate constraint, and 20 (9.1%) as having high constraint. The distribution of RASCI categories is presented in Table 5 and Figure 5.

## 4. Discussion

In the present study, we performed a comprehensive CT-based morphometric evaluation of the OW–FN surgical corridor by integrating OW dimensions, OW niche width, facial nerve proximity, and mastoid pneumatization status. The main finding was that increasing mastoid pneumatization was positively associated with OW width and FN–OW distance. This suggests that mastoid pneumatization may not merely represent an isolated mastoid characteristic, but may also reflect the broader spatial organization of the temporal bone. In addition, the observed relationship between OW morphology and FN proximity indicates that these parameters should not be evaluated as isolated linear measurements in preoperative radiological assessment. Accordingly, the proposed RASCI was developed as a preliminary radiological–anatomical scoring framework to stratify the potential spatial constraints of the OW–FN corridor. However, because this index was derived from CT measurements in radiologically normal temporal bones, its clinical utility requires prospective validation in surgical cohorts.

The morphometry of the OW has been demonstrated to be directly related to stapes surgery, prosthesis selection and trans-OW approaches. As demonstrated in previous anatomical studies, the size and shape of the OW vary considerably between individuals. This variability may have implications for stapedotomy, stapedectomy, ossicular reconstruction and implant design [6,7]. The present study examined the relationship between OW height and several parameters related to the corridor, including OW area, FNt-OW distance, FNm-OW distance and OW-FNm-FNt area. This finding is significant as the OW height may serve as an indicator of the vertical accessibility of the footplate region, which in turn may have an effect on the available working space for instrumentation. These findings support the view that OW morphology, particularly in anatomically narrow or borderline OW niche configurations, should be systematically evaluated on preoperative CT, in accordance with previous reports emphasizing the significance of OW niche morphology in stapes surgery [5,17].

The OW niche constitutes an additional clinically relevant segment of the surgical corridor. A narrow OW niche has the potential to restrict the visualisation of the footplate and the manoeuvrability of surgical instruments, particularly in instances where an overhanging or low-lying facial nerve is encountered. Parra et al. have indicated that the width and depth of the OW niche, in conjunction with the facial-promontory angle, are radiological parameters that correlate with the surgeon’s access to the footplate. These parameters may assist in predicting difficult stapes surgery [17]. In the present cohort, an association was observed between OW niche width and the distances between OW and FNt, OW and FNm, and OW, FNm and FNt. This finding suggests that the niche is not merely a local bony recess, but rather constitutes a component of a more extensive OW–FN spatial configuration. This lends support to the rationale for incorporating OW niche width in the RASCI. However, the individual discriminatory value of OW niche width should be interpreted with caution until it has been validated with intraoperative data.

The facial nerve’s relationship to the OW is of paramount importance to the safety of stapes and middle ear surgery. The location of the facial nerve in its tympanic segment is just superior to the OW, and it has been demonstrated that this can restrict access to the footplate, particularly in cases of dehiscence, prolapse or overhang. It has been demonstrated in previous studies that overhanging FN is visible on both high-resolution and ultra-high-resolution CT scans. The presence of overhanging FN has been shown to complicate footplate exposure and piston placement, which are processes that are typically undertaken separately [12]. Furthermore, clinical series of stapes surgery in patients with dehiscent or prolapsed facial nerve anatomy have demonstrated that surgical modification may be necessary, including altered prosthesis positioning or restricted nerve manipulation in selected [13]. In the present study, larger OW dimensions and wider OW niche widths were found to be associated with greater FN-OW distances. This suggests that OW morphology and FN proximity should be interpreted together rather than separately.

A salient finding of the present study was the correlation between the mastoid pneumatization and the OW-FN corridor. As the degree of mastoid pneumatization increased, so too did the distances between FNt–OW and FNm–OW. Concurrently, the area encompassing OW–FNm–FNt expanded. This finding lends further support to the hypothesis that mastoid pneumatisation may be a contributing factor. These findings lend support to the hypothesis that mastoid pneumatization may be associated with the spatial relationship of deeper temporal bone structures. Dai et al. previously reported that the relationship between mastoid pneumatization and the position of the mastoid segment of the facial nerve is complex and may be influenced by the development of the temporal bone and the adjacent vascular structures [1]. In addition, studies evaluating mastoid pneumatization on CT have shown that the extent of pneumatization is associated with surgical anatomy and may influence the position or vulnerability of structures such as the facial canal, sigmoid sinus, and facial recess [18,19,20].

The findings of the present study lend further support to the utilisation of sigmoid sinus-based mastoid pneumatization classification as a practical radiological marker. Han et al. [22] proposed a classification system that was predicated on the relationship between the extension of the mastoid air cells and the sigmoid sinus, as depicted on CT images. Subsequent studies have corroborated the hypothesis that the sigmoid sinus constitutes a valuable reference structure for the degree of temporal bone pneumatisation. Aladeyelu et al. [21] also proposed that the assessment based on the sigmoid sinus may be both simple and reproducible, particularly at the level of the lateral semicircular canal. This may assist otologists in evaluating the structures that may be involved to varying degrees of pneumatization. It is evident from the results of this study that the mastoid pneumatization grading method may offer insights into two distinct areas of research. Firstly, it provides valuable information regarding the development of the mastoid air cells. Secondly, it provides insights into the spatial characteristics of the OW–FN corridor.

The development of RASCI was predicated on the assumption that an integrated scoring approach may benefit the preoperative assessment of OW-related procedures. The utilisation of radiological parameters in preceding imaging-based studies has proven efficacious in the prediction of challenging surgical anatomy. Parra et al. proposed imaging criteria to predict the complexity of stapes surgery. As posited by Tang et al., a methodology for the identification of facial canal dehiscence through the utilisation of imaging scoring techniques on ultra-high-resolution CT scans has been proposed [16,17]. In this context, the RASCI differs from isolated morphometric measurements by combining OW morphology, OW niche width, mastoid pneumatization, and FN proximity into a single preliminary radiological–anatomical index. In the present cohort, the majority of temporal bones were categorised as exhibiting moderate radiological–anatomical constraint, whereas a smaller proportion demonstrated high constraint. This distribution indicates that radiologically normal temporal bones may still demonstrate variable degrees of anatomical restriction within the OW–FN corridor. However, it is important to note that the RASCI score should not be considered a validated clinical risk score at this stage. In contrast, it signifies a systematised radiological structure that has the potential to facilitate the standardisation of anatomical reporting and the generation of hypotheses for subsequent surgical validation.

Recent radio-clinical studies have further emphasized that preoperative HRCT may provide predictive information beyond the diagnosis of middle-ear pathology. In cochlear implantation, although surgical access is primarily directed toward the round window or cochleostomy rather than the oval window, radiological evaluation of the facial nerve and posterior tympanotomy corridor has been shown to predict surgical accessibility [23,24]. A recent HRCT-based classification of the vertical part of the facial nerve demonstrated a significant correlation with intraoperative round-window visibility and the need for additional surgical maneuvers during pediatric cochlear implantation [23]. Similarly, a prospective radioclinical scoring system for ordinary cochlear implantation showed that preoperative radiological parameters were strongly correlated with posterior tympanotomy difficulty and surgical duration, with the chorda-facial angle reported as one of the strongest predictors [24]. In stapes surgery, comparable predictive radiological approaches have also been proposed [25,26]. A prospective study evaluating the radiological incudo-stapedial angle showed that this parameter was significantly associated with the choice of stapedotomy technique, with obtuse or right angles generally permitting reversal-steps stapedotomy, whereas acute angles favored the traditional non-reversal technique [25]. Another radio-clinical study demonstrated that preoperative scutum length could predict oval window fossa accessibility during stapedotomy and the potential need for intraoperative scutum curettage [26]. Together, these studies support the concept that structured HRCT-based assessment of middle-ear anatomy can anticipate surgical exposure, technical difficulty, and operative strategy [23,24,25,26]. In this context, the present RASCI model similarly aims to provide a preliminary radiological–anatomical framework for evaluating the OW–FN corridor, particularly for oval-window-related middle-ear procedures.

The present study should not be interpreted as a re-description of isolated OW, FN, or stapes-region anatomy, as these structures have been extensively investigated in previous anatomical and radiological studies. Instead, the primary contribution of this study lies in the integrated evaluation of OW morphology, OW niche width, FN proximity, and mastoid pneumatization within a unified HRCT-based framework. In this context, mastoid pneumatization is not proposed as an independent determinant of surgical difficulty. Conversely, when integrated with local OW–FN corridor parameters, it can function as a component of a structured radiological–anatomical evaluation. Consequently, the proposed RASCI should be regarded as a preliminary and hypothesis-generating framework for describing OW–FN corridor configuration, rather than as a validated clinical predictor of operative difficulty or complications.

It should be emphasized that the present study did not identify pathological anatomical changes, as only radiologically normal temporal bones were included. Rather, the findings demonstrate measurable anatomical variations and spatial configurations within the OW–FN corridor. These variations included differences in OW width and height, OW niche width, FNt–OW and FNm–OW distances, and mastoid pneumatization grade. Therefore, the proposed RASCI should be interpreted as a framework for stratifying radiological–anatomical corridor configuration, rather than as a tool for detecting pathological middle-ear changes. From a clinical perspective, the correlation between greater mastoid pneumatization, larger OW dimensions, and increased FN–OW distances may facilitate the interpretation of the OW–FN corridor by radiologists and surgeons as part of the broader temporal bone configuration rather than as isolated measurements. In stapes surgery and other OW-related middle-ear procedures, a temporal bone with reduced OW height or width, a narrow OW niche, short FN–OW distances, and poor mastoid pneumatization may indicate a more spatially constrained operative field. Such a configuration may prompt a more meticulous review of preoperative HRCT images, careful assessment of possible facial nerve overhang or limited footplate exposure, selection of finer instruments, consideration of endoscopic assistance, or anticipation of modified surgical maneuvers. In revision procedures, where anatomical landmarks may be distorted or exposure may already be limited, the structured preoperative identification of a narrow OW–FN corridor may be particularly useful for surgical planning. Although cochlear implantation is primarily performed through the round window or cochleostomy rather than the oval window, the same principle applies to preoperative evaluation of middle-ear corridors, especially with respect to facial nerve position, posterior tympanotomy access, and round-window visibility. However, given that the present study did not encompass intraoperative difficulty grading or postoperative outcomes, these clinical implications must be regarded as preliminary and require validation in prospective surgical cohorts. A suggested clinical interpretation of the RASCI categories for preoperative HRCT review is summarized in Table 6.

### Limitations

The study is not without its limitations. First, this was a retrospective single-centre radiological investigation. Second, the study included only radiologically normal temporal bones; therefore, the findings may not be directly generalizable to patients with otosclerosis, chronic otitis media, congenital anomalies, or revision middle-ear surgery. A further important limitation is the absence of a diseased-ear comparison group. Thus, the present findings should be interpreted as a baseline radiological–anatomical assessment of normal temporal bones, and future comparative studies are required to determine whether RASCI categories differ between normal and diseased ears and whether they correlate with intraoperative difficulty. Third, the proposed RASCI was not correlated with intraoperative difficulty grading, operative time, technical modifications, postoperative hearing outcomes, or complication rates. Consequently, it should be regarded as a preliminary radiological–anatomical framework rather than a validated clinical prediction instrument. In addition, the RASCI thresholds and point weights were not derived from surgical outcome-based modelling; therefore, future studies should validate and, if necessary, recalibrate these cut-offs and weights using intraoperative difficulty, operative time, and postoperative outcome data. Finally, given that both ears of each patient were included, the potential interdependence between the right and left temporal bones may not have been fully eliminated. Future validation should ideally be performed in prospective surgical cohorts, including patients with otosclerosis, chronic otitis media, congenital anomalies, and revision middle-ear surgery. The correlation of RASCI categories with operative time, surgeon-rated difficulty scores, necessity for technical modification, postoperative hearing outcomes, and facial nerve-related or vestibular complications is recommended. The implementation of such studies would facilitate outcome-based recalibration of the current thresholds and point weights. In the event of validation, RASCI could also be incorporated into structured preoperative HRCT reporting templates with a view to standardising the radiological assessment of the OW–FN corridor prior to OW-related middle-ear procedures.

## 5. Conclusions

The CT-based evaluation of the OW–FN corridor demonstrated that mastoid pneumatization is associated with OW morphology and the spatial relationships between the FN and OW. It was established that larger OW-related dimensions, larger FN–OW distances, and a wider OW–FN corridor area were associated with greater mastoid pneumatization. This finding suggests that mastoid pneumatization may serve as a radiological indicator of a more general spatial organisation of the temporal bone. The proposed RASCI offers a preliminary radiological–anatomical framework to stratify potential OW–FN corridor constraints in the preoperative assessment. However, given that this index was derived from radiologically normal temporal bones and has not been tested against intra-operative outcomes, prospective surgical validation is required before its use as a clinical predictor of operative difficulty or complications can be justified.

## Figures and Tables

**Table 1:** Descriptive statistics results of oval window and facial nerve.

Parameters	N	Min–Max	Mean (mm) ± SD
OW width	220	0.2–5.2	2.41 ± 0.92
OW height	220	0.69–2.5	1.38 ± 0.36
OW niche width	220	0.22–1.6	1.03 ± 0.38
OW area (mm^2^)	220	0.49–3.7	2.86 ± 0.75
FNt-OW distance	220	0.37–1.5	0.93 ± 0.33
OW mastoid cell distance	220	2.13–4.2	3.44 ± 0.61
FNm-OW distance	220	0.9–3.99	2.78 ± 0.94
The area between FNm-FNt-OW (mm^2^)	220	8–34	14.00 ± 1.00

Min: Minimum, Max: Maximum, SD: Standard Deviation, OW: Oval window, FNt: Tympanic segment of the facial nerve, FNm: Mastoid segment of the facial nerve.

**Table 2:** Relation of OW height with parameters.

Parameters	OW Height Classification (mm)			*p*
	<0.8 (I)	0.8–1.4 (II)	>1.4 (III)	
OW width	0.98 ± 0.36	1.30 ± 0.67	1.52 ± 0.46	0.039 (I–III)
OW niche width	0.94 ± 0.29	1.08 ± 0.38	1.09 ± 0.42	0.62
OW area (mm^2^)	2.81 ± 0.26	2.88 ± 0.85	2.86 ± 0.77	0.986
FNt-OW distance	0.83 ± 0.31	0.95 ± 0.38	0.98 ± 0.33	0.062
OW-mastoid cell distance	2.83 ± 0.61	3.24 ± 0.59	3.50 ± 0.6	0.038 (I–III)
FNm-OW distance	2.13 ± 0.83	2.58 ± 0.98	2.88 ± 0.94	0.01 (I–III, II–III)
The area between FNm-FNt-OW (mm^2^)	9.00 ± 1.00	11.00 ± 2.00	17.00 ± 1.00	0.048 (I–III)

Test: Kruskal–Wallis test, *p* < 0.05, OW: Oval window, FNt: Tympanic segment of the facial nerve, FNm: Mastoid segment of the facial nerve.

**Table 3:** Relation of OW width with parameters.

Parameters	OW Width Classification (mm)			*p*
	<1.00 (I)	1.00–2.00 (II)	>2.00 (III)	
OW height	2.11 ± 0.67	2.51 ± 1.40	2.70 ± 0.86	0.000 (I–III, II–III)
OW niche width	1.11 ± 0.51	1.00 ± 0.33	1.10 ± 0.42	0.232
OW area (mm^2^)	2.16 ± 1.14	3.01 ± 0.47	3.21 ± 0.44	0.000 (I–III, II–III)
FNt-OW distance	0.91 ± 0.93	0.97 ± 0.29	0.98 ± 0.42	0.000 (I–III, I–II)
OW-mastoid cell distance	3.04 ± 0.79	3.54 ± 0.01	3.57 ± 0.82	0.000 (I–III, I–II)
FNm-OW distance	2.74 ± 0.04	2.79 ± 0.76	3.03 ± 1.02	0.038 (I–III, II–III)
The area between FNm-FNt-OW (mm^2^)	9 ± 5	11 ± 1	19 ± 2	0.000 (I–III, II–III)

Test: Kruskal–Wallis test, *p* < 0.05, OW: Oval window, FNt: Tympanic segment of the facial nerve, FNm: Mastoid segment of the facial nerve, FN: facial nerve.

**Table 4:** Correlation matrix of oval window, facial nerve distance, and corridor area parameters.

		1	2	3	4	5	6	7	8
OW height	r	1	0.361 **	0.243 **	0.587 **	0.370 **	0.235 **	0.245 **	0.307 **
	p	.	0	0	0	0	0	0	0
OW width	r		1	0.317 **	0.133 *	0.357 **	0.039	0.318 **	0
	p		.	0	0.047	0	0.56	0	0.996
OW niche width	r			1	0.246 **	0.889 **	0.248 **	0.450 **	0.431 **
	p			.	0	0	0	0	0
OW area (mm^2^)	r				1	0.318 **	0.417 **	0.09	0.460 **
	p				.	0	0	0.179	0
FNt-OW distance	r					1	0.278 **	0.534 **	0.447 **
	p					.	0	0	0
OW mastoid cell distance	r						1	0.192 **	0.678 **
	p						.	0.004	0
FNm-OW distance	r							1	0.194 **
	p							.	0.003
OW-FNm-FNt-area (mm^2^)	r								1
	p								.

OW: Oval window, FNt: Tympanic segment of the facial nerve, FNm: Mastoid segment of the facial nerve, FN: facial nerve (1: OW width; 2: OW height; 3: OW niche width; 4: OW area; 5: FNt–OW distance; 6: OW–mastoid cell distance; 7: FNm–OW distance; 8: OW–FNm–FNt area).: * indicates statistical significance at *p* < 0.05, and ** indicates statistical significance at *p* < 0.01. A dot (.) indicates that the *p*-value is not applicable because the variable is correlated with itself.

**Table 5:** Preliminary Radiological–Anatomical Surgical Corridor Index (RASCI) Categories.

		Frequency	Percent	Valid Percent	Cumulative Percent
Valid	Low radiological–anatomical constraint	47	21.4	21.4	21.4
	Moderate radiological–anatomical constraint	153	69.5	69.5	90.9
	High radiological–anatomical constraint	20	9.1	9.1	100.0
	Total	220	100.0	100.0	

**Table 6:** Suggested clinical interpretation of RASCI categories for preoperative HRCT review.

RASCI Category	Radiological–Anatomical Interpretation	Possible Preoperative Implication
Low constraint	Relatively favorable OW dimensions, wider OW niche, greater FN–OW distances, and/or well-pneumatized mastoid configuration	Routine structured HRCT review; standard OW-related surgical planning may be sufficient
Moderate constraint	Borderline OW dimensions or OW niche width, intermediate pneumatization, or moderately reduced FN–OW spatial relationship	Careful multiplanar HRCT review; attention to OW niche exposure, facial nerve proximity, and instrument maneuverability
High constraint	Narrow OW dimensions, narrow OW niche, short FN–OW distances, and/or hypopneumatized mastoid configuration	Anticipation of limited exposure; careful assessment for facial nerve overhang or restricted footplate access; consideration of finer instruments, endoscopic assistance, or modified surgical strategy depending on intraoperative judgment

## Data Availability

The data that support the findings of this study are available upon request from the corresponding author. The data are not publicly available due to privacy or ethical restrictions.

## Abbreviations

The following abbreviations are used in this manuscript:

RASCI	Radiological–Anatomical Surgical Corridor Index
HRCT	High-Resolution Computed Tomography
OW	Oval Window
FN	Facial Nerve
FNt	Tympanic Segment of the Facial Nerve
FNm	Mastoid Segment of the Facial Nerve
OWn	Oval Window Niche
